# Understanding the contribution of habitats and regional variation to long-term population trends in tricolored blackbirds

**DOI:** 10.1002/ece3.681

**Published:** 2013-07-22

**Authors:** Emily E Graves, Marcel Holyoak, T Rodd Kelsey, Robert J Meese

**Affiliations:** 1 Department of Environmental Science and Policy, University of California1 Shields Avenue, Davis, California, 95616; 2 Audubon California765 University Avenue, Suite 200, Sacramento, California, 95825

**Keywords:** *Agelaius tricolor*, agriculture, habitat switching, population trend, precipitation, tricolored blackbird

## Abstract

Population trends represent a minimum amount of information required to assess the conservation status of a species. However, understanding and detecting trends can be complicated by variation among habitats and regions, and by dispersal connecting habitats through source-sink dynamics. We analyzed trends in breeding populations between habitats and regions to better understand the overall dynamics of a species' decline. Specifically, we analyzed historical trends in breeding populations of tricolored blackbirds (*Agelaius tricolor*) using breeding records from 1907 to 2009. The species breeds itinerantly and ephemerally uses multiple habitat types and breeding areas, which make interpretation of trends complex. We found overall abundance declines of 63% between 1935 and 1975. Since 1980 overall declines became nonsignificant and obscure despite large amounts of data from 1980 to 2009. Temporal trends differed between breeding habitat types and were associated with regional differences in population declines. A new habitat, triticale crops (a wheat-rye hybrid grain) produced colonies 40× larger, on average, than other breeding habitats, and contributed to a change in regional distribution since it primarily occurred in a single region. The mechanism for such an effect is not clear, but could represent the local availability of foodstuffs in the landscape rather than something specific to triticale crops. While variation in trends among habitats clearly occurred, they could not easily be ascribed to source-sink dynamics, ecological traps, habitat selection or other detailed ecological mechanisms. Nonetheless, such exchanges provide valuable information to guide management of dynamic systems.

## Introduction

For populations, temporal trends in abundance represent an important type of information on which to base conservation and management decisions. It is therefore important to understand the causes of population trends, and reasons why such trends vary geographically, temporally or among species. While there are many natural and anthropogenic causes of sustained population declines (negative population trends), frequently identified factors include habitat loss and fragmentation (e.g., Virkkala 1991; Donovan and Flather 2002; Sirami et al. 2009), reduction in habitat quality (Benton et al. 2002), natural enemies (Schmidt 2003), harvesting (Fryxell et al. 1988), climate change (Winfield et al. 2010), non-native invasive species (Gurevitch and Padilla 2004), and alteration of disturbance regimes (Holmes and Sherry 2001). Nonetheless, temporal trends are sometimes complex to interpret and detect for reasons that include the limitations of statistical methods and the biology and physical structure of the study system. Perhaps the most challenging systems in which to understand population trends are those that include multiple habitat types and where movement patterns among different habitat areas are largely unknown – which is the case in many systems.

Spatial variation in demography and mixing of populations can arise from geographical variation (Morrison et al. 2010), spatial-scale dependence (Houlahan et al. 2000), and involvement of multiple connected habitat types (e.g., Virkkala 1991). Species showing habitat-specific demography may show variation in trends across habitats. Also, in source-sink systems (Pulliam 1988), regular dispersal among habitat types may blur habitat-specific trends. Similar problems of spatial scale dependence and effects of dispersal are expected from ecological traps (reviewed by Robertson and Hutto 2006) and species showing habitat selection behavior. For example, habitat preference might mask population trends if only preferred habitats were monitored and if these sites were buffered from population declines by immigration from less-preferred habitats (Rodenhouse et al. 1997). Trend detection and interpretation is also complicated for nomadic or itinerantly breeding species, which are expected to be especially likely to show switches among habitat types and regions. Such species also frequently show rapid population growth in response to favorable environmental conditions or food availability (Orians 1961), which would create naturally variable population sizes and these would be expected to hinder trend detection. Surprisingly there have been few analyses of trends that consider multiple habitat types and real-world complexities such as those listed above (although see Helle and Järvinen 1986; Rodenhouse et al. 1997).

Long-term population studies are especially valuable for their ability to identify population dynamic patterns. We investigated population trends spanning >100 years across a large portion of the geographic range for a colonially nesting, itinerantly breeding songbird of conservation concern. Tricolored blackbirds (*Agelaius tricolor*) are a striking example of a range-restricted colonial bird that has experienced a major decline in the last 80 years (e.g., Beedy and Hamilton 1999). However, the relative contribution of different factors to the decline is poorly known, including the effect of any changes in use of different types of breeding habitats. Likewise we do not know the extent of any decline during recent decades. Previous studies have tabulated population sizes but have not statistically analyzed population data (DeHaven et al. 1975; Beedy et al. 1991; Beedy and Hamilton 1997; Hamilton et al. 1999).

In this study we analyzed the most extensive dataset yet compiled for the species, comprising 2463 records of the sizes of breeding colonies. As the species are broadly dispersed in mixed species flocks during winter, breeding surveys are the most practical method to investigate population trends. We performed the first systematic statistical evaluation of trends for tricolored blackbirds to address five questions: (1) What is the magnitude of the overall decline, and is it continuing? (2) Do trends vary across regions? (3) Do trends vary among breeding habitat types? (4) Has there been a change in the net geographic distribution of the species? (5) Are changes in regional distribution linked to changes in habitats used for breeding? We use our findings to derive management and research recommendations.

### The study species

The most extensive reports of tricolored blackbird population status indicate range-wide breeding abundance declines of ∼89% between the 1930s and 1980s (Beedy et al. 1991). The species is the most colonial passerine in North America since the extinction of passenger pigeons (Bent 1958). Concentration of breeding in large colonies makes the species especially vulnerable to dramatic nesting failures (Beedy and Hamilton 1999; Cook and Toft 2005; Meese 2013). The species is largely endemic to California (>99% of birds), with small populations in adjacent states and Baja California, Mexico. Since the 1930s, over 90% of the individuals nested in wetlands of California's Central Valley (Neff 1937; Orians 1961; DeHaven et al. 1975). This area experienced wetland losses of greater than 90% between 1850 and 1980 (Frayer et al. 1989), and ∼99% loss of grassland habitats that are used for foraging by tricolored blackbirds (Beedy and Hamilton 1997). For tricolored blackbirds, Beedy et al. (1991) reported wetland loss and fragmentation as the principal reasons for decline. Yet, the species has also changed from predominantly breeding in freshwater cattail (*Typha* spp.) and bulrush (*Scirpus* spp.) marshes (Neff 1937) to increasingly include upland non-native and agricultural habitats as breeding sites (Beedy and Hamilton 1997). Such changes could complicate our interpretation and detection of population declines. Tricolored blackbirds also exhibit semi-nomadic behavior and itinerant breeding (Orians 1961; Hamilton 1998), making it hard to accurately assess overall status.

There have been several previous descriptions of tricolored blackbird populations. Neff (1937) recorded over 736,000 adults in 1934 in just eight counties, and during 5 years recorded 252 colonies in 26 counties, with the largest being over 300,000 birds. DeHaven et al. (1975) reported that populations declined by at least 50% between the 1930s and 1970s, with average annual counts in the 1970s of 133,000 birds. Beedy and Hamilton (1997) questioned this finding because the 1970s surveys did not include large breeding colonies in the southern San Joaquin Valley. Conversely, Beedy et al. (1991) reported continued declines since the work of DeHaven et al. (1975), with an annual average of 52,000 adult breeding birds per year in the 1980s. Finally, Hamilton et al. (1999) reported continued declines (since the 1970s) based on extensive surveys in the 1990s. Trends in abundance have not been systematically and statistically analyzed. It is possible that the decline has slowed since the 1970s because of breeding in nonnative upland habitats (Himalayan blackberry, *Rubus armeniacus*, and some cereal grain fields), from which Cook and Toft (2005) reported higher nesting success than from native marshes. Meese (2013) found that insect abundance in foraging habitats was correlated with reproductive success but that no such native versus nonnative breeding habitat effect on reproductive success was found. Meese's (2013) work distinguished breeding substrates where colonies nest from foraging habitats that were up to 9 km from colonies. Such a separation of habitat types is more detailed than most literature reports, and in the present article we refer to “breeding habitat” as the substrate in which nests are located.

Cited reasons for decline of tricolored blackbirds include loss of breeding and foraging habitats, pesticide usage, disturbance through agricultural harvesting, predation (e.g., by herons and egrets; Meese 2012), occasional deliberate poisonings with avicides to protect crops, and early 20th century market-harvesting of blackbirds (Neff 1937; Beedy et al. 1991; Beedy and Hamilton 1997). More broadly, agricultural intensification has been linked to songbird declines in farmland (Donald et al. 2001; Benton et al. 2002; Wretenberg et al. 2007). There is a substantial scope for conflict between tricolored blackbirds and agriculture. This is because a large proportion of tricolored blackbirds occur in California's agriculturally intensive Central Valley (DeHaven et al. 1975) and recent occurrence of large colonies in triticale fields (a wheat-rye hybrid, and an acronym of *Triticum* [wheat] and *Secale* [rye]) that are frequently at risk of being destroyed during harvest while nests are still active. Increased occurrence in the San Joaquin Valley (southern part of the Central Valley) is anecdotally reported to be linked to a decline in the dairy industry in Southern California, raising the possibility that there was movement of birds away from Southern California. Hence, farming practices may have large effects on tricolored blackbirds.

The tricolored blackbird receives legal protection by the Migratory Bird Treaty Act. A petition to list the species as threatened or endangered under the U.S. Endangered Species Act was declined in 2006 because of inadequate information (Federal Register 2006). However, it has been classified as a nongame species of management concern since 1995 (U.S. Fish and Wildlife Service 1995) and California Species of Special Concern since 1990. Additionally, the Bureau of Land Management listed it as a sensitive species since 1999 (Bureau of Land Management 2006), and it has been on the IUCN red list of endangered species since 2006 (IUCN 2011).

## Materials and Methods

### Data sources

Breeding censuses are often the most practical way to record widespread changes in population status of songbirds (e.g., see Link and Sauer 1998, for the North American Breeding Bird Survey). We compiled data and used those from the public Tricolored Blackbird Portal (http://tricolor.ice.ucdavis.edu/), an online database for recording observations of tricolored blackbird breeding colonies, including their locations, habitat used for nesting, occupancy, estimates of numbers of breeding birds, and records of observations of color-banded birds. We entered historical records from literature sources into the Tricolored Blackbird Portal. Our analyses used records from 1907 to 2009, reflecting that we initiated our analyses late in 2009. The portal was also used for participants in the 2008 statewide census to enter their observations (Kelsey 2008). Of the 2463 total records in the database, 29.2% (*n* = 719) were from published manuscripts and 70.8% (*n* = 1744) were from gray-literature reports. These reports primarily represent volunteer-based statewide surveys sponsored by the USFWS conducted in 1994, 1997, 1999, 2000, 2001, 2005, and 2008 (Hamilton et al. 1995; Beedy and Hamilton 1997; Hamilton et al. 1999; Hamilton 2000; Humple and Churchwell 2002; Sloat 2005; Hamilton and Meese 2006; Kelsey 2008). See “Additional data entry procedures” section in Appendix for additional details that relate to subsets of the data. All plant species names are given in Appendix Table A1.

### Statistical analyses

Reported locations of tricolored blackbirds that were not nesting locations were excluded from analyses. The number of birds per breeding record was used as the most accurate available metric of bird abundance. We also examined total abundance within regions, although we note that such an index is subject to variation caused by sampling effort. The most comprehensive tabulation of historical population abundances, by Beedy et al. (1991), found the main declines to occur between the “1930s” and “1970s”. We used the years 1935 and 1975 to be equivalent to and facilitate comparison with these earlier reports. To evaluate recent population trends, we selected 1980 data onward since it allows 30 years of data since that time (1980–2009 inclusive), giving a reasonable sample size for time-series analyses of trends and to encompass the time period after which Beedy et al. (1991) reported population declines. In addition, the 1980s represent a period when some large-scale changes in geographical distribution were observed, with regional formation of large colonies in the southern Central Valley of California that may have been attributed to increases in crops like triticale and growth of the dairy industry. The choice of 1980 as a cut-off rather than another year (e.g., 1975, 1985) did not change the significance of our results for recent trends. For sites with multiple visits per year we used the peak recorded abundance per site as the best available estimate of abundance.

Abundance data were natural logarithmically transformed to meet assumptions of normality. All statistical tests were performed in *Statistica 6.0* (Statsoft Corp., Tulsa, Oklahoma). To test for trends we used linear regression to test the slope of ln(birds per breeding record) versus year number, both in an overall test using all data and in a separate test using just data after 1980. Durbin–Watson tests on residuals tested for temporal autocorrelation. As a check on data consistency through time we also looked at the CV of abundance (see “Population variability” section in Appendix).

General linear models (GLMs) were used to test for differences in temporal trends in ln(abundance) among geographic regions and common breeding habitats in separate tests. Attempting to combine these analyses to maximize the comparability of results gave us an either/or choice: general linear models could contain region × year or habitat × year, and the significance depended on what was already in the model; also models with region × year or habitat × year had a ΔAIC of <2, suggesting no justifiable difference in support for each model. The interaction between region or habitat and year was used to test for differences in slopes, representing the strength of temporal trends. Model parameter estimates and standard errors were used to identify means that varied for factors significant at *P* < 0.05. As a measure of effect size, the proportion of variance explained by explanatory variables was compared using partial eta-squared (*h*^2^) = (SS_effect_)/(SS_effect_ + SS_error_). Finally, we also reran the statistics using linear mixed effects models using function lmer (in package lme4) in R (R Development Core Team 2011) to check that the results held up if year and/or region or location were used as random factors to account for the correlated error structure in the data, and also using Poisson errors rather than Gaussian errors: none of these refinements changed the results obtained, and we therefore present the simpler results that we originally obtained. We used G-tests to test whether the proportion of records in different habitats varied among regions; in these analyses we excluded habitats with <5% of records across all regions.

## Results

### Population status and the extent of declines

The database contained 1964 records of breeding or nonbreeding birds, from 1183 different sites in 46 counties. It included 501 additional records from known prior locations where no birds were recorded that were not used in our analyses. Breeding was recorded at 74% (880) of the 1182 recorded sites (breeding was unclear at one site). There were 243 sites with multiple records of breeding birds. Hence, 28% of the 880 breeding sites were used in multiple years, and this likely represents a minimum estimate because frequently data were lacking about whether sites were revisited to check continued breeding.

**Figure 1:**
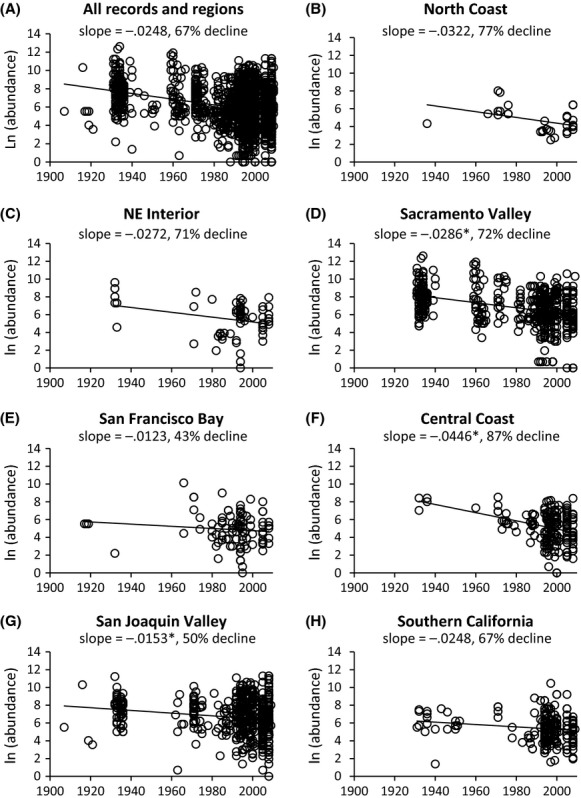
Trends in ln(abundance = colony size) for (A) all breeding colonies and colonies within geographic regions (B–H; see Fig. 2C for region definitions). The lines show results of linear regressions detailed below for A, and in Table 1 for B–H. For all years in A, ln(abundance) = 55.62 − 0.02479 × year (e.g., 2009); *t*_675_ = 9.0, *P* < 0.001; adjusted *r*^2^ = 0.11 (11% of variation); residuals autocorrelation was weak, that is serial autocorrelation coefficient of 0.27. (B–H) Compared with all other regions the Central Coast had larger colonies in earlier years but declined more rapidly, and the San Joaquin Valley showed smaller colonies in early year that declined less rapidly. Regression slopes of ln(abundance) of breeding birds versus year are given, and in B–H asterisks indicate a difference *P* ≈ 0.05 between Southern California (as indicative of a representative trend—compare A and H) and the region indicated by an asterisk. The % decline in mean breeding colony size (number of birds) from 1935 to 1980 is also given as a measure of historical decline.

Overall the number of birds per record (colony size) declined significantly and substantially from 1935 to 1975 (Fig. 1A; these years are chosen to be consistent with reports in the literature – see Discussion). Mean breeding colony size was estimated as 2103 birds in 1935, compared with 780 birds in 1975 (from regression in Fig. 1A). Hence, mean abundance per breeding site declined by 63% from 1935 to 1975, but much variation remains unexplained (Fig. 1A; from the regression *r*^2^ = 11%). In contrast, we did not find evidence for a decline in average colony size from 1980 onwards. A regression of ln(abundance) versus year for breeding colonies from 1980 onwards was not significant despite having 1572 degrees of freedom (*t*_1572_ = −1.60, *P* = 0.11). A power test showed that with α = 0.05 and sample size the same as that after 1980 the regression of ln(abundance) versus year provided power of β = 0.82 for a slope (trend magnitude) the same as that observed prior to 1980. To detect a slope of just 20% of the observed pre-1980 magnitude, these data provide power of β = 0.62. Hence, power was reasonable even to detect low rates of decline.

### Regional declines and habitat types

**Figure 2:**
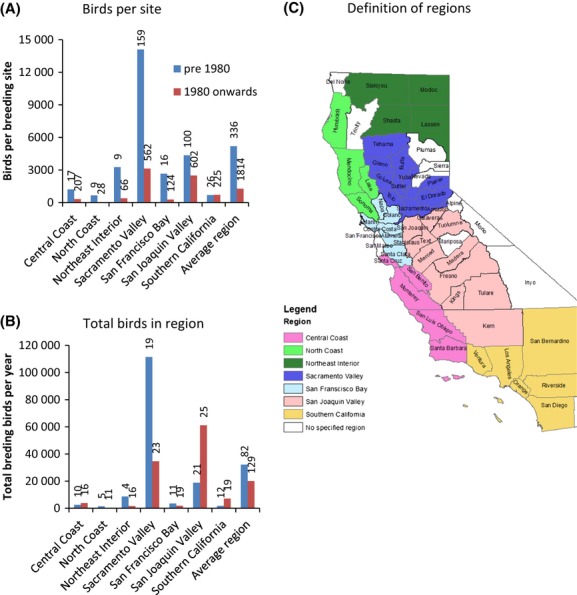
(A) Average of maximum number of breeding birds per colony per year, and (B) total number of breeding birds recorded per year in each region during the periods before 1980 (blue bars) and from 1980 to 2009 (red bars). Numbers of records are shown above each bar. (C) The location of geographical regions and the counties that comprise these regions, with shadings indicated by the key to regions on the map. White (no specified region) indicates that no breeding tricolored blackbirds were recorded during the entire study period.

**Figure 3:**
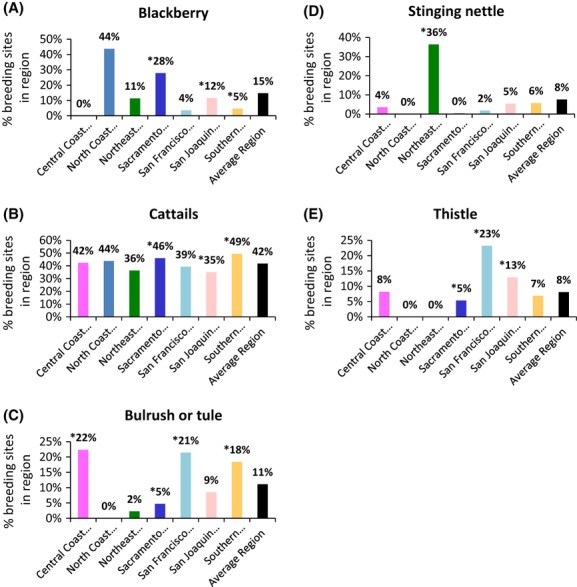
Percentage of breeding sites with the most common habitats within each region. An asterisk above a bar indicates that the habitat differed from the all-region average frequency for that habitat at *P* < 0.05 in a G-test. Overall G-tests checked for significance across regions (protecting alpha) and then G-tests for heterogeneity were performed among regions. No G-tests were conducted for the North Coast because there were only 16 breeding sites in total.

**Figure 4:**
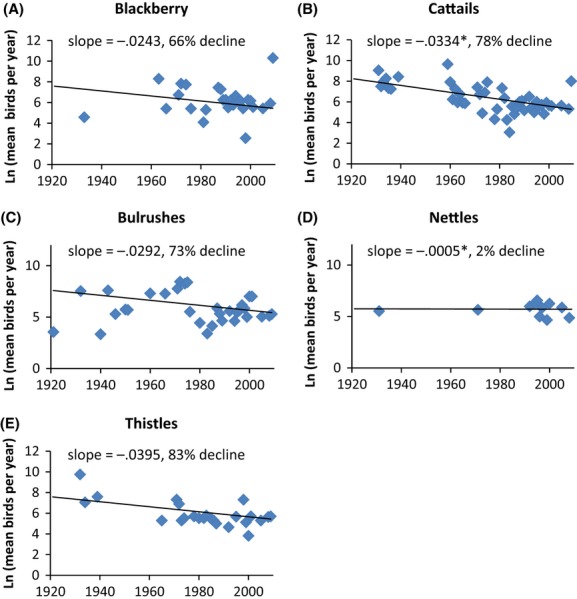
Trends in numbers of breeding birds in different common habitat types. Lines show linear regressions from the analysis detailed in Table 2. Regressions are identical for A, C, and E (Table 2). Each point shown is an annual mean; for instance there were three records that comprised the single point for nettles in 1971, and hence the outlying data points are less severe than they look in the figure.

As described in more detail in the following paragraphs, we found geographical variation in tricolored blackbird breeding population trends, as shown by the average size of breeding colonies (Fig. 1; Table 1). Like the size of breeding colonies, total populations changed substantially, as exemplified by comparing pre-1980 data to those from 1980 onwards (Fig. 2A and B). There were also different frequencies of breeding habitat types across regions (Fig. 3) and there were some corresponding differences in temporal trends among habitat types (Fig. 4, Table 2). Regions are defined in Figure 2C. A caveat for all of our analyses is that region explained only 20.8% of variation in trends in average breeding colony size (Table 1), and the comparable figure for habitat type was only 14.5% of the variation (Table 2); this variation is also shared by terms in the general linear models that we used to examine trends, so that the amount of variation in trends explained by differences among regions or habitats is quite small (see *h*^2^ values Tables 1 and 2).

**Table 1:** Results of a general linear model testing for differences in ln(abundance) versus year of reporting for breeding records among geographical regions

	SS	df	MS	*F*	*P*	*h*^2^
Intercept	159.05	1	159.05	47.56	0.001	0.030
Region	52.98	6	8.83	2.64	0.015	0.010
Year	128.59	1	128.59	38.46	0.001	0.024
Region × Year	52.92	6	8.82	2.64	0.015	0.010
Error	5286.73	1581	3.34			

Parameter type	Region	Parameter	SE	*t*	*P*
Intercept	Southern California	54.921	7.963	6.90	0.001
Difference in intercept	Central Coast	39.277	19.601	2.00	0.045
Difference in intercept	North Coast	13.907	32.651	0.43	0.670
Difference in intercept	Northeast Interior	4.584	20.727	0.22	0.825
Difference in intercept	Sacramento Valley	8.469	9.274	0.91	0.361
Difference in intercept	San Francisco Bay	−25.641	19.921	−1.29	0.198
Difference in intercept	San Joaquin Valley	−17.755	9.990	−1.78	0.076
Slope	Southern California	−0.0248	0.0040	−6.20	0.001
Difference in slope	Central Coast	−0.0198	0.0098	−2.02	0.044
Difference in slope	North Coast	−0.0074	0.0164	−0.45	0.651
Difference in slope	Northeast Interior	−0.0024	0.0104	−0.23	0.821
Difference in slope	Sacramento Valley	−0.0038	0.0047	−0.81	0.417
Difference in slope	San Francisco Bay	0.0125	0.0100	1.25	0.211
Difference in slope	San Joaquin Valley	0.0095	0.0050	1.89	0.059

The whole model adjusted *R*^2^-value was 20.8%. The first part of the table reports standard ANOVA table values and the second part reports parameter values. Effect size is given as the proportion of variance explained by explanatory variables, partial eta-squared (*h*^2^) = (SS_effect_)/(SS_effect_ + SS_error_). For Southern California the intercept and slope are shown, whereas differences from these values and significance of these differences are given for other habitats.

**Table 2:** Results of a general linear model testing for differences in ln(abundance) among the most frequently occurring habitats versus year of reporting for breeding records

	SS	df	MS	*F*	*P*	*h*^2^
Intercept	165.8	1	165.8	46.60	0.001	0.036
Habitat	42.7	4	10.7	3.00	0.018	0.009
Year	132.1	1	132.1	37.12	0.001	0.029
Habitat × Year	42.7	4	10.7	3.00	0.018	0.009
Error	4589.4	1290	3.6			

Parameter type	Habitat	Parameter	SE	*t*	*P*
Intercept	Blackberry	54.17	7.94	6.83	0.001
Difference in intercept	Cattails	18.19	8.84	2.06	0.040
Difference in intercept	Bulrush or tule	9.66	13.94	0.69	0.489
Difference in intercept	Stinging nettle	−47.50	17.43	−2.73	0.007
Difference in intercept	Thistle	30.20	16.44	1.84	0.066
Slope	Blackberry	−0.0243	0.0040	−6.09	0.001
Difference in slope	Cattails	−0.0091	0.0044	−2.06	0.040
Difference in slope	Bulrush or tule	−0.0049	0.0070	−0.70	0.486
Difference in slope	Stinging nettle	0.0238	0.0087	2.72	0.007
Difference in slope	Thistle	−0.0152	0.0082	−1.84	0.066

The whole model adjusted *R*^2^-value was 14.5%. Effect size is given as the proportion of variance explained by explanatory variables, partial eta-squared (*h*^2^) = (SS_effect_)/(SS_effect_ + SS_error_). The first part of the table reports standard ANOVA table values and the second part reports parameter values. For Himalayan blackberry colonies the intercept and slope are shown, whereas differences from these values and significance of these differences are given for other habitat types.

All regions showed negative trends in average breeding colony size through time but there was some variation between regions in the rate of decline. In 1935 the Central Coast had 72% larger colonies than the average across all regions but subsequent to this these sites declined 80% more rapidly than colonies in other regions (Table 1; Fig. 1F). Compared with other regions (Fig. 1B–E, G, and H), San Joaquin Valley colonies were 32% smaller (*P* = 0.08) in 1935 (except the Central Coast), but declined at a 38% slower rate after this (*P* = 0.06; Table 1, Fig. 1G). Other geographical regions did not vary from one-another in the trends observed. Figure 2 summarizes net changes in both the numbers of birds per breeding colony (Fig. 2A) and the total annual number of breeding birds per year in each region from 1935 to 1980 (Fig. 2B). Sacramento Valley and San Joaquin Valley both stand out in having had a large number of records relative to other regions prior to 1980. For the Sacramento Valley the typical statewide negative trend in average breeding colony size reported above (Fig. 1A and D) was accompanied by a large decline in the total regional breeding population per year in that region (Fig. 2B). Conversely, the San Joaquin Valley showed both a less severe (and marginally significant) trend in average colony size compared to other regions (Fig. 1G) and the total regional breeding population actually increased from pre-1980 to 1980 onwards. After 1980 the San Joaquin Valley on average held more breeding birds than any other region (Fig. 2B), whereas prior to 1980 the Sacramento Valley held far larger populations than any other region.

The frequency of breeding habitat types varied significantly between regions (Fig. 3; *G*_40_ = 93.8, *P* < 0.001; “Use of different breeding habitat types” section in Appendix gives additional detail on frequencies of use of all habitat types statewide). Breeding colonies in cattail marshes were more frequent than the statewide average in the Sacramento Valley and Southern California, and less frequent than the statewide average in the San Joaquin Valley (Fig. 3B). Triticale is not shown in Figure 3, but all records were from the San Joaquin Valley and just to the north of there in Sacramento County. Bulrush sites were more frequent than the average across regions in the Central Coast, San Francisco Bay and Southern California, and less frequent than average in the Sacramento Valley (Fig. 3C). The Sacramento Valley had far more sites with Himalayan blackberry than the statewide average, and the San Joaquin Valley and Southern California had less blackberry sites than the statewide average (Fig. 3A). Stinging nettle sites were disproportionately frequent in Northeast Interior breeding sites (Fig. 3D), as was thistle in San Francisco Bay region (Fig. 3E).

Temporal trends in mean colony size also varied between habitats (Table 2; Fig. 4). In general, colony size declined through time except for colonies in native stinging nettles, which showed no temporal change in size (Table 2; Fig. 4D). It is surprising that population sizes declined even within existing marsh habitats (Fig. 4B and C). It is not clear whether such a decline represents a reduction in area of the breeding habitat occupied or whether it is attributable to another factor such as changes within foraging habitat or quality of breeding habitat. Colonies in cattails were 34% larger in the early years of records compared to those in blackberry, bulrush, and thistle, but declined 38% more rapidly (Table 2; Fig. 4B). Colonies in Himalayan blackberry, bulrush, and thistle did not differ significantly from one-another in size or rate of decline (Table 2; Fig. 4A, C, and E). Small sample size and all records being relatively recent prevented us from analyzing temporal trends in triticale, which had 14 breeding records in the database from 13 locations between 1999 and 2009. Average colony size was markedly larger in triticale (mean = 24,871 birds, SE = 7697 birds) than other habitats (mean = 639 birds, SE = 1.1, *n* = 193; Student's *t*_205_ = 3.04, *P* < 0.01).

## Discussion

### Population status and the extent of declines

The substantial breeding population declines of tricolored blackbirds that we found from 1935 to 1980 (Fig. 1A) are consistent in magnitude with earlier reports, but much variation remains unexplained. We found a 63% decline in breeding abundance (mean colony size) from 1935 to 1975, whereas Beedy et al. (1991 – from data in their Table 1) amassed data showing a 76% decline in colony size between the “1930s and 1970s” (which we took as 1935 to 1975 seeking equivalence). We did not find evidence for a decline in average colony size since the 1970s despite having good sample sizes and reasonable statistical power. This is contrary to Beedy et al. (1991), whose data (their Table 1) show a 62% decline between the 1970s and 1980s. Kyle and Kelsey (2011) also reported a 34% decline in breeding bird numbers in 2011 compared with 2008, despite the 2011 survey including 72% more sites than the 2008 survey (Kelsey 2008). However, it is hard to interpret such short-term trends because the survey data show high interannual variability (“Population variability” section in Appendix). Ultimately, more years of surveys with a similar sampling effort to the statewide breeding surveys are needed. Appropriately, Kyle and Kelsey (2011) recommended a triennial range-wide survey and an annual survey in three especially important counties (Merced, Kern, and Tulare), all of which are within the San Joaquin Valley.

Unlike average colony size, total (summed) population size across all breeding sites was (not surprisingly) strongly related to the total number of sites sampled. Consistent with this problem of sample size dependency, Beedy et al. (1991) reported an 89% decline in total breeding populations from the 1930s to the 1980s, whereas we found a 69% decline in this time period in total breeding populations. Because of the sensitivity of total population size to sampling effort we do not recommend using total population size as a metric of population status for this species.

Habitat loss is stated as the reason for decline in breeding populations (Beedy et al. 1991). However, the direct loss of breeding sites cannot explain why colony size declined within existing marshes (Table 2; Fig. 4), many of which are protected (e.g., National Wildlife Refuges) and are the same localities since the 1930s. Wetland loss has also slowed in recent years because of protection and mitigation resulting from the 1977 amendment of the Clean Water Act and other measures (e.g., Dahl 2006). Changes in habitat quality are generally harder to evaluate. Likely quality changes have occurred in foraging habitats through agricultural intensification leading to disturbance from harvesting and increased pesticide usage, which diminishes insect populations required for breeding (Beedy et al. 1991; Beedy and Hamilton 1997; Benton et al. 2002). Meese (2013) also found that over a 6-year period (2006–2011) over 40 colonies had chronically low reproductive success, and reproductive success was correlated with insect abundance in foraging grounds; the determinants of insect abundance are largely unknown. There are also specific incidences of quality change that are clear, for instance the draining of marshes and senescence of marsh vegetation (Meese 2008). Historically, almost all wetlands in the Central Valley were managed for wintering migratory birds, with little attention to or capacity for managing spring wetlands when tricolored blackbirds would use these wetlands. In recent years some sites in National Wildlife Refuges (e.g., Kern, Merced) and some marshes owned by duck clubs have been managed specifically for tricolored blackbird breeding, however, these represent very few sites relative to the habitat requirements for this species.

### Regional declines and habitat types

We found substantial changes in breeding populations in different regions and breeding habitat types (Figs. 1 and 4). These regional declines corresponded to trends in different breeding habitats, with four caveats. First, the total amount of variation in breeding bird abundances explained by habitat, region and time variables was only 14.5% to 20.8%, and some variation was shared by model terms (see *h*^2^-values in Tables 1 and 2); hence while there is an effect it is not strong. Second, we cannot, based on correlational data alone, distinguish whether habitats drive regional differences or vice versa (or whether an unrelated factor drives both). Third, our statistical analyses could not fully include triticale since it is represented by only a small number of records, so the effect is quantified and discussed but cannot be directly compared with the results in Tables 1 and 2. Fourth, the data analyzed are for the presence of colonies: we do not have data from consistent monitoring or habitat areas regardless of occupancy by breeding tricolored blackbirds.

Prior to 1980 the Sacramento Valley held the largest number of birds, whereas from 1980 onwards the San Joaquin Valley supported the largest total breeding populations of tricolored blackbirds. We believe two factors are involved in the slow decline in average colony size in the San Joaquin Valley and growth of total breeding populations (Table 1, Figs. 1G and 2). First, colonies in triticale were all within the San Joaquin Valley (or Sacramento County), all during the last 20 years, and they were >40× larger than colonies in other habitats during this period. Second, cattail sites and blackberry sites were uncommon in the San Joaquin Valley. Central coast colony-size declines resulted from four early records (Fig. 1F), and three of these came from cattails in which declines were rapid (Table 2, Fig. 4B). The decline of Sacramento Valley breeding populations consisted of both a reduction in average colony size (Fig. 2A) and the total breeding population (Fig. 2B), and hence the number of sites occupied. Given that many of the marsh (cattail and bulrush/tule) sites in this region are in wildlife refuges it is surprising that such colonies declined in average size. However, increased management for wintering waterfowl may have altered the marshes from their historical conditions. Possibly the observed declines indicate that something other than breeding habitat per se affects breeding populations, and this might be something such as insects in foraging habitat (e.g., Meese 2013), or be associated with climate change.

Overall the observed trends in breeding populations in different habitats are consistent with regional changes we observed (albeit subject to the caveats listed above). Our observed changes in populations in different regions and habitats are consistent with Beedy et al. (1991) and Cook and Toft (2005). Himalayan blackberry sites showed slower declines in average colony size than other habitats, and cattail sites declined in average colony size more rapidly than other habitats. Similarly, the high proportion of cattail sites in the Sacramento Valley was coincident with more rapid declines in this area than the statewide average. Differences among habitats clearly contributed to a change in net geographic distribution, as well as altering overall temporal trends. In our cases we do not directly know what aspect of habitat alters the demography or movements of tricolored blackbirds, whether it is breeding habitat or foraging habitat for instance (Meese 2013). A few other studies have related bird population trends to habitat types. Virkkala (1991) tied regional population trends in Finnish birds to habitat types, and found that habitats that experienced the greatest loss or fragmentation showed the largest population declines. Seoane and Carrascal (2008) found that trends in Spanish birds varied among habitat types, and Wretenberg et al. (2007) showed that Swedish birds occupying agricultural habitats were most likely to show population declines.

The long-term changes in the proportions of birds in different habitats (Fig. 2) could result either from birds moving among habitats (within or across years), or from the long-term differences in fitness playing out. Itinerancy likely contributes to change in the types of habitats used. Hamilton et al. (1995) reported that site occupancy was short-lived, 15% of sites being occupied for 2–3 years, and 26% being occupied for at least 4 years (the number of 1 year colonies was unclear). Meese (2011) also reported 84 new colony locations discovered from 2005 to 2011. These figures indicate some selection of new breeding colony locations on a yearly basis. Additionally, birds may breed at several different sites within a year (Beedy and Hamilton 1999) but the majority of database records represent the first spring breeding.

An important piece of biology is that we do not know whether tricolored blackbirds are philopatric to more permanent habitats, whether the same individuals regularly move among habitats, or whether there is more permanent emigration to different habitat types. In the closely related red-winged blackbird, source-sink dynamics were demonstrated with exchange of individuals between rural source (low predation) and urban sink (high-predation) habitats (Vierling 2000). For tricolored blackbirds it is unclear whether exchanges represent source-sink dynamics (Howe et al. 1991), ecological traps (Robertson and Hutto 2006), habitat selection, or buffer populations. In buffer populations, individuals in low-quality habitats represent individuals excluded by territoriality (density dependence) from high-quality habitats, but such individuals would move to higher quality habitats if populations in them declined, thereby buffering such populations from decline (Rodenhouse et al. 1997). While these details remain obscure, the ability of exchanges among habitats to modify range-wide and regional population trends is clearer.

### Conservation recommendations

The variety of breeding habitats used by tricolored blackbirds and the ephemerality of breeding site occupancy in many habitats makes it difficult to disentangle the factors behind overall population trends. Himalayan blackberry and thistles also represent nonnative invasive species, so we are left with a conundrum of needing to protect areas of an invasive species to protect tricolored blackbird colonies (Cook and Toft 2005). Furthermore, over 50% of the breeding population in any given year was within relatively few triticale farm field colonies, requiring protection of these in at least the short term for conservation of this species. Itinerant breeding and the potential for movement of birds among habitats lead to several recommendations: (1) Monitor breeding, protect colonies, and analyze population trends in a full range of habitats; even sink habitats may contribute to reproductive output (Howe et al. 1991). (2) Institute and reinforce conservation measures that allow colonies to regularly occur in new areas and successfully complete breeding, including in annual crops such as triticale (discussed further below). (3) Work with private landowners where agricultural field colonies occur to create alternative, sustainable natural habitats outside of grain fields. (4) To conduct further studies of habitat quality and breeding success (e.g., Meese 2013; K. Weintraub, unpubl. data) to ascertain whether there are long-term trends in these characteristics and quantify long-term habitat-specific demography in relation to both breeding habitats and surrounding foraging habitats. The effects of landscape context of breeding colonies also need further study (Meese 2013), to determine the extent to which the habitat used for nesting versus the foraging habitat influences breeding success. (5) Given the ephemerality of some colonies, construct a stochastic metapopulation model and obtain empirical data on colony longevity and productivity to evaluate the long-term contribution to persistence and total population size in different habitats.

Only one of 13 colony locations in triticale was recorded as lasting for >1 year, compared with 28% of colonies in other habitats. However, we have observed that tricolored blackbirds may move to adjacent habitat areas when an originally occupied area was unavailable (e.g., due to crop rotation). We know that in the case of triticale there is more frequent reuse of sites when the habitat was replanted (from records after data were extracted in 2009), but replanting was infrequent. Because of the large size of colonies, triticale is an important habitat for tricolored blackbirds but is vulnerable to harvesting of the crop prior to young birds fledging (e.g., Kyle and Kelsey 2011). Although protected by the Migratory Bird Treaty Act (Federal Register 2006), the conservation of colonies in ephemeral habitats is entirely voluntary, and some colonies are conserved while others are lost each year (Meese 2008).

## Conclusion

Despite recent increases in sampling of tricolored blackbirds through statewide breeding surveys, post-1990 trends are unclear, or based on very few years of data (Kyle and Kelsey 2011). The range-wide population decline has not occurred uniformly among habitats and regions: a relatively recent agricultural crop (triticale) has supported large breeding populations in the San Joaquin Valley and resulted in an increased proportion of birds being within this region compared to records prior to the 1980s. However, this habitat is ephemeral and carries with it a high risk of failure through harvesting. Understanding overall population trends requires understanding variation among habitats and regions.

## Appendix

### Methods

#### Additional data entry procedures

Historical observations from Beedy et al. (1991) were often estimates and rough descriptions of colony locations. To standardize data entry, we used the following pragmatic procedures. When only the name of the city was provided for a colony location, the coordinates for that colony were entered as the center of that city as given by Google Earth (Google 2011, Version 5.1.3533.1731, http://www.google.com/earth/index.html). When only a city/town name and direction (no distance) were provided, the coordinates for that colony were entered as 1 mile (1.6 km) in the specified direction from the city center. If the number of birds observed was given as “hundreds of individuals,” the minimum bird count was entered as 200, the maximum bird count as 300, and the best estimate as 250. If the number of birds observed was given as “several hundred individuals,” the minimum bird count was entered as 300, the maximum bird count as 400, and the best estimate as 350. If the number of birds observed was given as the number of pairs, the number of birds observed was entered as 2.5 times the number of pairs observed (Payne 1969). If the number of birds observed was given as the number of nests, the number of birds observed was entered as 1.5 times the number of nests observed, reflecting that this species is polygynous and colonies typically have twice the number of females as males (Payne 1969). All observations from Sloat (2005) were entered into the database with the observation date as 24 April of the specified year, as day and month were not provided.

Records of nonbreeding birds in the same location as a breeding colony during the breeding season were excluded from breeding records. Records of flying birds at uncertain locations were not entered. When possible, records from the 1994, 1997, 1999, 2001, and 2005 surveys were cross-checked against records from the California Natural Diversity Database (CNDDB; Bittman 2001). The CNDDB also contained observation data from before and after the survey dates, which were entered when sufficient information was available. The CNDDB provided more accurate location descriptions and coordinates than did the survey reports in some cases, which allowed for correction of uncertain locations from the survey reports Bittman 2001.

### Results

#### Population variability

Variability of abundance (CV) among sites within each year and year number were positively correlated (1919–2009, excluding single-record years; *r*_50_ = 0.37, *P* = 0.003). The increase in the CV was also strongly correlated with sample size (*r*_50_ = 0.69, *P* < 0.001), indicating that the increased variability was more controlled by sampling effort than population dynamics.

Encouragingly, interannual variability in colony size for colonies with multiple years of recorded breeding (CV of abundance across years within sites) did not change with year of recording (*r*_241_ = 0.0003, *P* = 0.997). We interpret this as meaning that early and recent censuses are of similar reliability, and that conditions did not change sufficiently to alter variation in year-to-year abundance within breeding sites.

#### Use of different breeding habitat types

Across all years the dominant breeding habitat was cattails, which represented 48% of breeding records and 65% of breeding birds (Table 3). Next most important for breeding numbers was triticale with 9% of birds, but only 1% of records because of the large size of colonies in triticale. Bulrushes (or “tules”) contained 7% of breeding birds and 9% of records. Himalayan blackberry accounted for 6% of breeding birds and 11% of records, and thistles for 5% of birds and 9% of records. Unknown breeding habitats (missing data in the original publication) and willows each accounted for 3% of records and 2% of breeding birds. Stinging nettles comprised 1% of birds but 5% of records. Other breeding habitat types were less frequently used (Table 3).

**Table A1:** Frequencies of records in different habitat types

Habitat	Total		Breeding		Nonbreeding	
		
	Records (%)	Total birds (%)	Records (%)	Total birds (%)	Records (%)	Total birds (%)
Cattails (*Typha* spp.)	400 (34%)	2,848,874 (53%)	326 (48%)	1,843,704 (65%)	74 (14%)	1,005,170 (43%)
Unknown	209 (18%	238,137 (5%)	19 (3%)	74,968 (2%)	190 (35%)	163,169 (7%)
Blackberry^1^	157 (13%)	648,137 (12%)	72 (11%)	175,518 (6%)	85 (16%)	472,619 (20%)
Bulrush or tule (*Schoenoplectus* spp.)	95 (8%)	380,706 (7%)	63 (9%)	202,550 (7%)	32 (6%)	178,156 (8%)
Thistles^2^	83 (7%)	227,486 (4%)	59 (9%)	142,850 (5%)	24 (4%)	84,636 (4%)
Stinging nettle (*Urtica dioica*)	47 (4%)	65,263 (1%)	32 (5%)	19,000 (1%)	15 (3%)	46,263 (2%)
Grassland^3^	36 (3%)	8085 (0.2%)	0 (0%)	0 (0%)	36 (7%)	8085 (0.3%)
Grain fields						
Triticale	14 (1%)	437,300 (8%)	8 (1%)	261,650 (9%)	6 (1%)	175,650 (7%)
Rice paddy	13 (1%)	8027 (0.2%)	5 (1%)	3150 (0.1%)	8 (2%)	4877 (0.2%)
Barley	5 (0.4%)	15,540 (0.3%)	1 0.1%)	4000 (0.1%)	4 (1%)	11,540 (1%)
Wheat	6 (0.4%)	78,775 (2%)	6 (1%)	45,500 (2%)	0 (0%)	33,275 (1%)
Other grain fields^4^	4 (0.3%)	6625 (0.1%)	1 (0.1%)	6000 (0.2%)	3 (1%)	625 (0.03%)
Agricultural fields						
Pasture	22 (2%)	37,801 (1%)	0 (0%)	0 (0%)	22 (4%)	37,801 (2%)
Mustard (*Brassica* spp.)	18 (2%)	106,667 (2%)	6 (1%)	65,250 (2%)	12 (2%)	41,417 (2%)
Feedlot	6 (1%)	3713 (0.1%)	0 (0%)	0 (0%)	6 (1%)	3713 (0.2%)
Alfalfa	5 (0.4%)	5300 (0.1%)	1 (0.1%)	1000 (0.03%)	4 (1%)	4300 (0.2%)
Other ag. fields^5^	3 (0.2%)	65,600 (1%)	1 (0.1%)	65,000 (2%)	2 (0.4%)	600 (0.03%)
Trees/Orchards						
Willows (*Salix* spp.)	26 (2%)	70,984 (1%)	23 (3%)	51,079 (2%)	3 (1%)	19,905 (1%)
Riparian trees	4 (0.3%)	8050 (0.2%)	0 (0%)	0 (0%)	4 (1%)	8050 (0.3%)
Tamarisk	2 (0.2%)	2787 (0.1%)	2 (0.3%)	2787 (0.1%)	0 (0%)	0 (0%)
Other trees/orchards^6^	10 (1%)	12,948 (0.2%)	2 (0.3%)	2200 (0.1%)	8 (2%)	10,748 (1%)
Shrubs and herbs						
Giant reed (*Arundo donax*)	5 (0.4%)	5651 (0.1%)	2 (0.3%)	3900 (0.1%)	3 (1%)	1751 (0.1%)
Atriplex or salt bush	7 (1%)	6536 (0.1%)	7 (1%)	4536 (0.2%)	0 (0%)	2000 (0.1%)
Others shrubs/herbs^7^	1 (1%)	47,565 (1%)	0 (0%)	0 (0%)	1 (0.2%)	47,565 (2%)
Other habitats						
Marsh	1 (0.1%)	1050 (0.02%)	0 (0%)	0 (0%)	1 (0.2%)	1050 (0.04%)
Wildflower field	1 (0.1%)	450 (0.01%)	0 (0%)	0 (0%)	1 (0.2%)	450 (0.02%)

1 Himalayan (*Rubus armeniacus*) 155 records of total (not breeding/nonbreeding), brambles 1 record, California blackberry (*Rubus ursinus*) 1 record.

2 Milk thistle (*Silybum marianum*) 48, bull thistle (*Cirsium vulgare*) 35.

3 Grassland 26, grazed grassland 4, mowed field 2, tall grass 2, wet grassland 1, Sudan grass 1.

4 Wheat silage 2, grain field 1, silage 1.

5 Lettuce (*Lactuca* spp.) 1, plowed field 1, tomato field 1.

6 Button willow 1, buttonbush 1, desert olive 1, eucalyptus 1, silver poplar 1, fruit tree 1, lemon orchard 1, orange grove 1, almond orchard 1.

7 Wild rose 2, Baccharis 1, mallow (*Malva sylvestris*) 1, wild raspberry 1, mulefat (*Baccharis viminea*) 1.

Representing contemporary patterns, from 1980 onward, 29% of breeding birds were recorded in cattails, 21% in triticale, 13% in Himalayan blackberry, 7% were in unknown habitat types, 5% in bulrush, 5% in prickly lettuce (*Lactuca serriola*), 4% in wheat, 4% in thistle, 3% in mustard, 3% in willows, 1% in stinging nettles, 1% in saltbush, and <1% in alfalfa, barley, giant reed, citrus groves, rice paddy, tamarisk, and wild rose.
